# Sex Differences in Associations Among Obesity, Metabolic Abnormalities, and Chronic Kidney Disease in Japanese Men and Women

**DOI:** 10.2188/jea.JE20150208

**Published:** 2016-08-05

**Authors:** Masaru Sakurai, Junji Kobayashi, Yasuo Takeda, Shin-Ya Nagasawa, Junichi Yamakawa, Junji Moriya, Hiroshi Mabuchi, Hideaki Nakagawa

**Affiliations:** 1 Department of Epidemiology and Public Health, Kanazawa Medical University, Kahoku, Ishikawa, Japan; 1 金沢医科大学医学部公衆衛生学; 2 Department of General Internal Medicine, Kanazawa Medical University, Kahoku, Ishikawa, Japan; 2 金沢大学医学部総合内科学; 3 Kanazawa Medical Association, Kanazawa, Japan; 3 金沢市医師会; 4 Department of Lipidology, Kanazawa University Graduate School of Medical Science, Kanazawa, Japan; 4 金沢大学脂質研究講座; 5 Medical Research Institute, Kanazawa Medical University, Kahoku, Ishikawa, Japan; 5 金沢医科大学総合医学研究所

**Keywords:** abdominal obesity, chronic kidney disease, metabolic syndrome, sex differences, 腹部肥満, 慢性腎臓病, メタボリックシンドローム, 性差

## Abstract

**Aims:**

The present study aimed to investigate relationships among abdominal obesity, metabolic abnormalities, and the prevalence of chronic kidney disease (CKD) in relatively lean Japanese men and women.

**Participants and methods:**

The participants included 8133 men and 15 934 women between 40 and 75 years of age recruited from the government health check-up center in Kanazawa City, Japan. The prevalence of abdominal obesity, high blood pressure, dyslipidemia, and high fasting plasma glucose levels were assessed according to the Japanese criteria for metabolic syndrome. The estimated glomerular filtration rate (eGFR) was calculated using the modified Modification of Diet in Renal Disease equation for the Japanese population, and participants with an eGFR <60 mL/min/1.73 m^2^ and/or proteinuria were diagnosed with CKD.

**Results:**

Overall, 23% of males and 14% of females met criteria for CKD. Having more numerous complicated metabolic abnormalities was significantly associated with a higher odds ratio (OR) of CKD for men and women, irrespective of abdominal obesity. However, there was a sex difference in the OR of CKD for obese participants without metabolic abnormalities, such that abdominal obesity without metabolic abnormalities was significantly associated with a higher OR for men (multivariate-adjusted OR 1.63; 95% confidence interval [CI], 1.16–2.28) but not for women (OR 1.01; 95% CI, 0.71–1.44).

**Conclusions:**

The present findings demonstrated that obesity without metabolic abnormalities was associated with a higher risk of CKD in men but not women in a relatively lean Japanese population.

## Abstract (ja)

**目的：:**

欧米人と比較し肥満の少ない日本人において、肥満・代謝異常と慢性腎臓病（CKD）との関連を横断的に検討し、その性差を明らかにすることを目的とした。

**対象と方法：:**

対象はK市の特定健診を受診した40-75歳の男性8,133名、女性15,934名。日本人のメタボリックシンドロームの判定基準を用いて腹部肥満および代謝異常（血圧高値、脂質異常、血糖高値）を判定した。日本人のGFR推算式により求めたeGFR低下（60 ml/min/1.73m^2^未満）またはタンパク尿陽性をCKDと判定した。ロジスティック回帰分析を用いて、各代謝異常の有無・合併数におけるCKD有病のオッズ比（OR）を求めた。

**結果：:**

男性の23%、女性の14%にCKDを認めた。男女とも、肥満の有無にかかわらず、代謝異常の合併数が増加するとCKD有病ORは上昇した。代謝異常のない肥満者のCKD有病には性差を認め、男性では肥満のみで有意なCKD有病リスクの増加を認め（多変量調整OR 1.63, 95%信頼区間1.16-2.28）、女性では肥満のみでは有意なCKDリスクの増加は認めなかった（OR 1.01, 95%信頼区間0.71-1.44）。

**結論：:**

代謝異常を合併しない肥満者において、男性ではCKD有病リスクが有意に高かったが、女性では有意な上昇は認めなかった。

## INTRODUCTION

The increasing number of patients with end-stage renal disease (ESRD) has been recognized as a major global public health problem.^1^ In 2002, the National Kidney Foundation (NKF) proposed a definition for chronic kidney disease (CKD) that encompasses a wide range of kidney diseases.^2^ CKD is not only a precursor of ESRD but has been identified as a risk factor for cardiovascular disease (CVD) in its early stages.^3^^,^^4^

Obesity is a representative risk factor of CKD^5^^–^^7^ because the hemodynamic and hormonal changes caused by excess weight and the accumulation of abdominal fat may increase the risk for CKD.^8^^–^^10^ It has also been shown that obesity-related metabolic abnormalities, such as hypertension,^11^^,^^12^ dyslipidemia,^13^^,^^14^ and diabetes mellitus,^13^^,^^15^ affect the progression of CKD. However, the prevalence of obesity among Asian individuals is relatively low,^16^ and it is not clear whether obesity itself or the metabolic abnormalities caused by obesity have a stronger effect on the progression of CKD.

Previous studies from our research group have demonstrated that the degree of obesity is closely associated with metabolic abnormalities in Japanese men, but that the associations between abdominal obesity and obesity-related metabolic abnormalities are not strong in Japanese women.^17^^,^^18^ While sex differences may exist in the association between abdominal obesity and CKD, few studies have separately evaluated the effects of obesity on the development of CKD in men and women in Asian populations, and the studies that have done so have produced controversial results.^5^^,^^6^ Furthermore, these studies used body mass index (BMI) as a marker of obesity rather than waist circumference, which may more closely related to abdominal adiposity.

Thus, the present cross-sectional study aimed to evaluate whether there are sex differences in the relationships among abdominal obesity, obesity-related metabolic abnormalities, and the prevalence of CKD in a general Japanese population.

## METHODS

### Participants

The participants included in the present study were recruited from the government health check-up center in Kanazawa City in central Japan. This specific health check-up and guidance program is provided for national health insurance subscribers living in Kanazawa City by the city’s National Health Insurance program. In 2008, a total of 25 139 residents in this area (8259 men and 16 880 women) between 40 and 75 years of age received a health check-up (response rate, 30%). Of the 25 139 potential participants, 1072 (4%) were excluded for various reasons: 1059 due to missing data (969 for missing serum creatinine [Cr] levels, 86 for a missing urine test, and four for missing waist-circumference measurements) and 13 due to severe CKD (estimated glomerular filtration rate [eGFR]: <15 mL/min/1.73 m^2^). Ultimately, 24 067 participants (8133 men and 15 934 women) were enrolled in the present study.

### Data collection

The health examinations, which were performed by physicians and/or nurses at clinics belonging to the Kanazawa Medical Association, included a medical history, physical examination, anthropometric measurements, and measurements of plasma glucose, glycated hemoglobin (HbA1c), and serum lipid levels. Height was measured without shoes to the nearest 0.1 cm using a standiometer; weight was measured to the nearest 0.1 kg using a standard scale, with participants not wearing shoes and wearing only light clothing; and BMI was calculated as weight/height squared (kg/m^2^). Blood pressure was measured after participants had rested for 5 minutes in a seated position. The standardization of these measurements was accomplished by following the guidelines for the specific health check-up and guidance program issued by the Ministry of Health, Labour and Welfare.

A questionnaire was used to identify voluntary health-related behaviors, including alcohol consumption and smoking, and an additional self-administered questionnaire was used to collect information regarding medical histories of hypertension, dyslipidemia, and diabetes. The prevalence of abdominal obesity, high blood pressure, dyslipidemia, and high plasma glucose levels were assessed according to the Japanese criteria for metabolic syndrome^19^^,^^20^: abdominal obesity was defined as a waist circumference ≥85 cm for men and ≥90 cm for women; high blood pressure was defined as systolic blood pressure ≥130 mm Hg, a diastolic blood pressure ≥85 mm Hg, or the use of antihypertensive medication; dyslipidemia was defined as serum triglyceride levels ≥150 mg/dL (fasting) or ≥200 mg/dL (postprandial), high-density lipoprotein (HDL)-cholesterol levels <40 mg/dL, or the use of medication for dyslipidemia; and glucose intolerance was defined as a fasting plasma glucose level ≥110 mg/dL, a postprandial plasma glucose level ≥140 mg/dL, a HbA1c level ≥6.0% (based on the National Glycohemoglobin Standardization Program), or the use of an anti-diabetic medication.

### Renal function tests

Urinary proteins levels were examined using a dipstick test, and eGFR was calculated based on serum Cr (sCr) levels and age using the following estimation equation, which was derived from insulin clearance data in a Japanese population^21^:

$$\begin{array}{l} \text{eGFR}(\text{mL/min/}1.73m^{2})\\ \;=194\times {\text{sCr}}^{-1.094}\times {\text{age}}^{-\text{0}.\text{287}}(\times 0.739\text{ for women}). \end{array}$$

In accordance with NKF guidelines,^2^ CKD was defined based on the presence of renal dysfunction, which was considered to be present if the eGFR was <60 mL/min/1.73 m^2^ and/or if the participant had dipstick urinary protein scores of ≥1+.

### Statistical analysis

The odds ratios (ORs) and 95% confidence intervals (CIs) for the prevalence rates of low eGFR (<60 mL/min/1.73 m^2^), proteinuria, and CKD for each component of metabolic syndrome were calculated using logistic regression analyses. The ORs were adjusted for age (continuous), smoking status (non-smoker or current smoker), alcohol consumption (nondrinker, occasional drinker, or everyday drinker), and the components of metabolic syndrome (abdominal obesity, high blood pressure, dyslipidemia, and glucose intolerance). Next, the association between the number of complicated metabolic abnormalities (high blood pressure, dyslipidemia, and glucose intolerance) and the prevalence of renal function was evaluated according to the presence of abdominal obesity. The difference in blood pressure among the groups according to the number of complicated metabolic abnormalities with/without obesity was evaluated using analyses of variance, and post-hoc testing was used for multiple comparisons. All statistical analyses were performed using the Statistical Package for the Social Sciences (IBM SPSS statistics version 22.0; IBM Corporation, Armonk, NY, USA). A *P*-value <0.05 was considered to indicate statistical significance.

### Ethical considerations

Written informed consent was not obtained from the participants. The Kanazawa Medical Association approved the design of the present study and ensured that individuals were not identifiable by providing linkable anonymous data to the researchers. The Institutional Review Committee of Kanazawa Medical University for Ethical Issues approved the present study.

## RESULTS

The characteristics of the study participants are presented in Table 1. The mean age at baseline was 67.1 years for men and 64.7 years for women, the mean BMI was 23.4 kg/m^2^ for men and 22.5 kg/m^2^ for women, and the mean eGFR was 73.8 mL/min/1.73 m^2^ for men and 76.2 mL/min/1.73 m^2^ for women. Overall, 50.7% of men and 20.8% of women exhibited abdominal obesity, and the prevalence rates of low eGFR, proteinuria, and CKD were 16.9%, 15.0%, and 22.7% for men, respectively; corresponding rates for women were 11.3%, 4.0%, and 14.3%.

**Table 1.** Characteristics of study participants

	Men (*n* = 8133)	Women (*n* = 15 934)
Mean (SD) age, years	67.1 (6.5)	64.7 (8.1)
Mean (SD) body mass index, kg/m^2^	23.4 (2.9)	22.5 (3.3)
Mean (SD) waist circumference, cm	84.9 (8.2)	82.2 (9.7)
Abdominal obesity^a^	50.7	20.8
High blood pressure^a^	68.8	57.9
Dyslipidemia^a^	42.2	38.1
High plasma glucose^a^	31.0	17.7
Current smoker	24.3	6.4
Alcohol consumption		
Non-drinker	32.6	72.0
Occasional	17.8	17.0
Everyday	49.6	10.9
Low eGFR	16.9	11.3
Proteinuria	15.0	4.0
Chronic kidney disease	22.7	14.3

eGFR, estimated glomerular filtration rate; SD, standard deviation.

Data are presented as percentages, unless otherwise noted.

^a^Abdominal obesity, high blood pressure, dyslipidemia, and high plasma glucose levels were defined in accordance with the definition of metabolic syndrome for the Japanese population.

Associations between the markers of renal damage and each component of metabolic syndrome are shown in Table 2. Abdominal obesity was associated with low eGFR, proteinuria, and CKD independent of other metabolic abnormalities among men and women. Of the four components of metabolic syndrome, dyslipidemia was most strongly associated with low eGFR and CKD in men and women. High blood pressure was also significantly associated with low eGFR, proteinuria, and CKD in men and women. High plasma glucose was associated with proteinuria, but not with low eGFR, after adjusting for other metabolic abnormalities, and it was not associated with CKD in men.

**Table 2.** Odds ratios of the presence of low eGFR, proteinuria, and chronic kidney disease for abdominal obesity and metabolic abnormalities in Japanese men and women

	Men			Women		
	
	χ^2^	OR^b^	95% CI	χ^2^	OR^b^	95% CI
Low eGFR						
Abdominal obesity^a^	10.4	1.23	(1.08–1.39)	21.9	1.32	(1.17–1.48)
High blood pressure^a^	22.7	1.41	(1.22–1.63)	7.5	1.17	(1.05–1.30)
Dyslipidemia^a^	62.8	1.64	(1.45–1.86)	26.5	1.31	(1.18–1.45)
High plasma glucose^a^	4.1	0.88	(0.77–1.00)	0.0	1.01	(0.89–1.14)
Proteinuria						
Abdominal obesity^a^	8.8	1.28	(1.09–1.50)	13.9	1.40	(1.18–1.68)
High blood pressure^a^	62.2	2.36	(1.90–2.92)	49.4	2.01	(1.66–2.45)
Dyslipidemia^a^	14.7	1.37	(1.17–1.60)	11.4	1.34	(1.13–1.58)
High plasma glucose^a^	66.4	1.93	(1.65–2.26)	26.4	1.62	(1.35–1.94)
Chronic kidney disease						
Abdominal obesity^a^	20.9	1.29	(1.16–1.44)	28.1	1.33	(1.20–1.47)
High blood pressure^a^	51.4	1.59	(1.40–1.81)	29.3	1.32	(1.20–1.46)
Dyslipidemia^a^	61.5	1.55	(1.39–1.73)	30.3	1.30	(1.19–1.43)
High plasma glucose^a^	2.9	1.10	(0.98–1.24)	4.6	1.13	(1.01–1.26)

CI, confidence interval; eGFR, estimated glomerular filtration rate; OR, odds ratio.

^a^Abdominal obesity, high blood pressure, dyslipidemia, and high plasma glucose levels were defined in accordance with the definition of metabolic syndrome for the Japanese population.

^b^ORs were adjusted for age, smoking status, alcohol consumption, and the four metabolic components (abdominal obesity, high blood pressure, dyslipidemia, and high plasma glucose).

**Figure 1.**
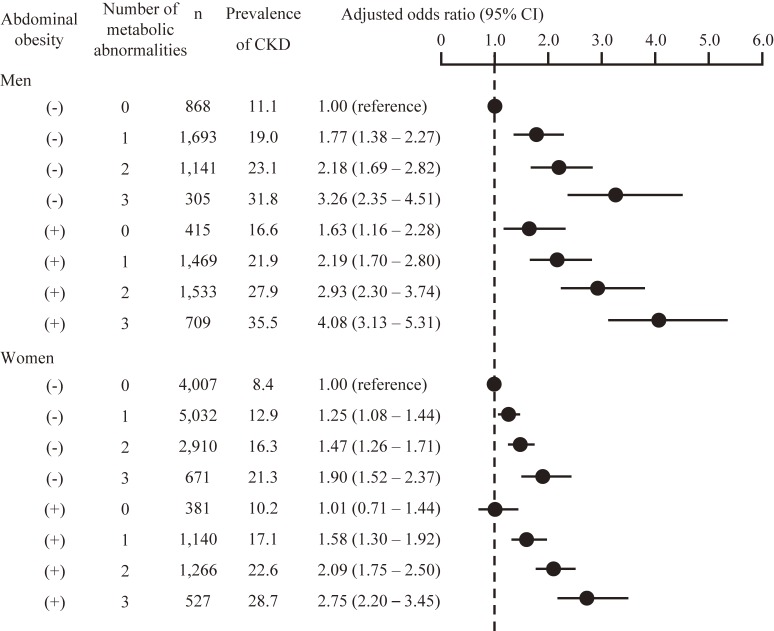
Prevalence of abdominal obesity, number of complicated metabolic abnormalities, and the odds ratio of chronic kidney disease in Japanese men and women. Odds ratios were adjusted for age, smoking status, and alcohol consumption. CI, confidence interval; CKD, chronic kidney disease.

**Figure 2.**
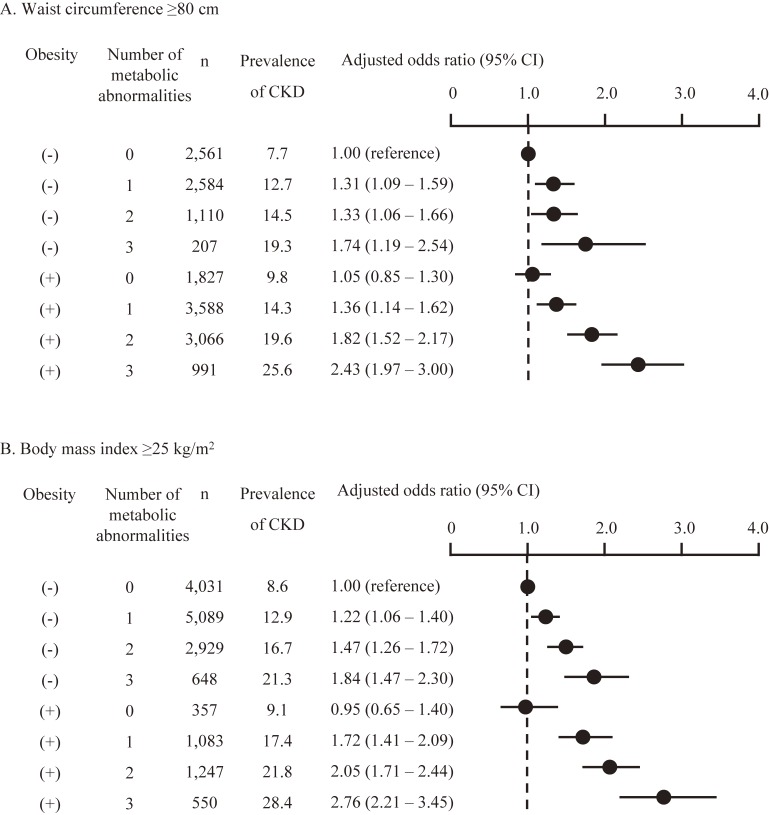
Prevalence of obesity, number of complicated metabolic abnormalities, and the odds ratio for the presence of chronic kidney disease in Japanese women. Obesity was defined by (A) the cut-off point for waist circumference according to the definition of metabolic syndrome for Asian women (80 cm), and (B), the BMI cut-off point of 25 kg/m^2^. Odds ratios were adjusted for age, smoking status, and alcohol consumption. CI, confidence interval; CKD, chronic kidney disease.

Next, the ORs of low eGFR, proteinuria (Table 3), and CKD (Figure 1) by the number of complicated metabolic abnormalities were evaluated according to the presence of obesity. Having numerous complicated metabolic abnormalities was associated with a higher OR for renal dysfunction, regardless of sex or the presence of abdominal obesity, but the effects of the interaction between abdominal obesity and the number of complicated metabolic abnormalities on the association with CKD were not significant. The interaction between sex and the number of complicated metabolic abnormalities with/without obesity was not significant (*P* = 0.304). However, male participants with abdominal obesity without metabolic abnormalities showed a higher risk of CKD, whereas abdominal obesity without metabolic abnormalities was not associated with a higher risk of CKD in women (Figure 1). Among the participants without metabolic abnormalities, systolic blood pressure was significantly higher in obese compared to non-obese women (mean [standard deviation] 116.1 [9.1] mm Hg vs 112.9 [10.1] mm Hg, *P* < 0.001), but was not higher in obese men than in non-obese men (mean [standard deviation] 116.2 [8.6] mm Hg vs 113.7 [9.8] mm Hg, *P* = 0.123). Diastolic blood pressure did not differ between non-obese and obese men and women. Even after adjusting for systolic blood pressure, the ORs for the presence of CKD were similar to the results of Figure 1 (data not shown). Obesity without metabolic abnormalities was not associated with a higher risk of CKD in women, even when a cut-off point for waist circumference of 80 cm was used (based on the definition of abdominal obesity for Asian women) or when a BMI ≥25 kg/m^2^ was used to diagnose obesity (Figure 2).

**Table 3.** Prevalence of abdominal obesity, number of complicated metabolic abnormalities, and odds ratios of renal dysfunction in Japanese men and women

Abdominal obesity	Number of metabolic abnormalities	Men				Women			
	
		*n*	Prevalence	OR^a^	(95% CI)	*n*	Prevalence	OR^a^	(95% CI)
Low eGFR									
(−)	0	868	8.4	1.00	(reference)	4007	6.8	1.00	(reference)
(−)	1	1693	15.0	1.80	(1.36–2.37)	5032	10.3	1.17	(1.00–1.37)
(−)	2	1141	17.2	1.98	(1.48–2.65)	2910	13.0	1.32	(1.11–1.56)
(−)	3	305	22.6	2.64	(1.83–3.82)	671	15.2	1.44	(1.12–1.85)
(+)	0	415	13.3	1.70	(1.17–2.49)	381	8.9	1.05	(0.72–1.54)
(+)	1	1469	16.5	2.08	(1.56–2.75)	1140	13.7	1.42	(1.14–1.76)
(+)	2	1533	20.4	2.60	(1.98–3.42)	1266	18.2	1.84	(1.51–2.23)
(+)	3	709	24.1	3.12	(2.31–4.22)	527	22.2	2.23	(1.74–2.85)
Proteinuria									
(−)	0	868	3.1	1.00	(reference)	4007	1.9	1.00	(reference)
(−)	1	1693	5.8	1.91	(1.23–2.95)	5032	3.3	1.66	(1.25–2.19)
(−)	2	1141	9.6	3.24	(2.10–4.99)	2910	4.3	2.14	(1.59–2.87)
(−)	3	305	16.1	5.80	(3.54–9.48)	671	8.9	4.56	(3.18–6.53)
(+)	0	415	3.4	1.08	(0.56–2.09)	381	2.1	1.05	(0.50–2.19)
(+)	1	1469	7.8	2.60	(1.69–4.00)	1140	4.4	2.19	(1.52–3.17)
(+)	2	1533	11.0	3.77	(2.48–5.72)	1266	6.9	3.49	(2.52–4.82)
(+)	3	709	20.3	7.73	(3.04–11.85)	527	11.2	5.88	(4.08–8.46)

CI, confidence interval; eGFR, estimated glomerular filtration rate; OR, odds ratio.

^a^ORs were adjusted for age, smoking status, and alcohol consumption.

## DISCUSSION

The present large-scale cross-sectional study of Japanese men and women found that abdominal obesity, diagnosed according to the definition of metabolic syndrome for the Japanese population, was associated with the prevalence of CKD independent of the presence of metabolic abnormalities. Further, having more numerous complicated metabolic abnormalities was associated with a higher prevalence of CKD in men and women. There was a sex difference in the association between the risk of CKD in participants with obesity but without metabolic abnormalities, with a higher risk of CKD in men but not women; however, the interaction between the sexes was not significant.

A previous prospective Japanese study demonstrated that a higher BMI is an independent risk factor for the development of ESRD in men but not in women,^22^ which is in partial accordance with our data. In contrast, the presence of obesity has also been associated with a higher risk of Stage I or II CKD in men and women.^5^ In the present study, abdominal obesity was associated with CKD in men and women when the associations were adjusted for the presence of other metabolic abnormalities. However, abdominal obesity also exhibited close associations with the presence of metabolic abnormalities, so the use of statistical models including both the presence of abdominal obesity and metabolic abnormalities might have been inappropriate due to multicollinearity. Therefore, stratification was employed to account for the influence of metabolic abnormalities on the association between abdominal obesity and CKD. The use of this method allowed for the clarification of the impact of obesity without metabolic abnormalities on the presence of CKD.

A number of studies have reported sex differences in the progression of renal diseases. For example, men are at a higher risk of CKD and tend to develop ESRD earlier in life than women,^23^^–^^25^ and these sex differences may be caused, at least in part, by the influence of testosterone and other sex hormones on the risks for proteinuria and glomerular sclerosis.^26^ Thus, variations in the levels of sex hormones may influence sex differences in the association between obesity and CKD. Sex differences in fat distribution may also affect the association between obesity and CKD. Our research group found that waist circumference does not effectively predict the existence of metabolic abnormalities in relatively lean Japanese women.^17^^,^^18^ Adipocytokines that are associated with visceral obesity, such as leptin and adiponectin, are associated not only with the pathogenesis of metabolic syndrome but also with that of CKD.^27^^–^^29^ Due to the strong influence of subcutaneous fat, but not visceral fat, on waist circumference,^30^ the presence of abdominal obesity would not be a useful marker of visceral adiposity in relatively lean Asian women. This may be one of the reasons that abdominal obesity without metabolic abnormalities was not associated with the presence of CKD in women in the present study.

Another possible cause of the sex differences observed in the association between obesity and CKD is the definition of abdominal obesity. In the present study, abdominal obesity was assessed according to the Japanese criteria for metabolic syndrome,^19^ which is a waist circumference ≥85 cm for men and ≥90 cm for women. These cut-off points differ from the cut-off points of 90 cm for men and 80 cm for women that are commonly used for Asian populations.^31^ It is possible that the cut-off point of 90 cm for waist circumference for women might be not appropriate for the detection of abdominal obesity; therefore, we further evaluated the association between obesity and CKD using the cut-off points of 80 cm for waist circumference and 25 kg/m^2^ for BMI in women. However, the associations using these different cut-off points were similar, which indicates that obesity itself was not associated with a higher risk of CKD in relatively lean Asian women.

One strength of this study was the large number of study participants recruited from a government health check-up center in a Japanese city. However, there were also several limitations to the present study. First, this was a cross-sectional study, and the use of a prospective design may provide additional information regarding the causal relationships between obesity and metabolic abnormalities and the progression of CKD. Another limitation was the fact that there was a low response rate to the government health check-up survey (30%). A person who is aware and conscious of his/her health may be more likely to respond to the health check-up, so the study population may have been healthier than the general population.

In conclusion, the present study found that obesity without metabolic abnormalities was associated with a higher risk of CKD in Japanese men but not in Japanese women. Thus, obesity without metabolic abnormalities should be recognized as a risk factor for CKD among men, but obesity itself may not influence the risk for CKD or metabolic abnormalities in relatively lean Japanese women.

## ONLINE ONLY MATERIAL

Abstract in Japanese.
