# Supplementary material for: Sex Differences in Associations Among Obesity, Metabolic Abnormalities, and Chronic Kidney Disease in Japanese Men and Women
Source: J Epidemiol. 2016 Aug 5;26(8):440–6. doi: 10.2188/jea.JE20150208 (PMC4967665; doi:10.2188/jea.JE20150208)
Supplement: Abstract in Japanese. [file je-26-440-s001.pdf]

日本人男女における，肥満・代謝異常と慢性腎臓病との関連の性差

櫻井　勝[1]、小林淳二[2]、竹田康男[3]、長澤晋哉[1]、山川淳一[2]、守屋純二[2]、馬渕宏[4]、中川秀昭[5]

[1] 金沢医科大学医学部公衆衛生学、[2] 金沢大学医学部総合内科学、[3] 金沢市医師会、[4] 金沢大学脂質研究講座、[5] 金沢医科大学総合医学研究所

目的：欧米人と比較し肥満の少ない日本人において、肥満・代謝異常と慢性腎臓病（CKD）との関連を横断的に検討し、その性差を明らかにすることを目的とした。

対象と方法：対象はK市の特定健診を受診した40-75歳の男性8,133名、女性15,934名。日本人のメタボリックシンドロームの判定基準を用いて腹部肥満および代謝異常（血圧高値、脂質異常、血糖高値）を判定した。日本人のGFR推算式により求めたeGFR低下（60 ml/min/1.73m^{2}未満）またはタンパク尿陽性をCKDと判定した。ロジスティック回帰分析を用いて、各代謝異常の有無・合併数におけるCKD有病のオッズ比（OR）を求めた。

結果：男性の23%、女性の14%にCKDを認めた。男女とも、肥満の有無にかかわらず、代謝異常の合併数が増加するとCKD有病ORは上昇した。代謝異常のない肥満者のCKD有病には性差を認め、男性では肥満のみで有意なCKD有病リスクの増加を認め（多変量調整OR 1.63, 95%信頼区間1.16-2.28)、女性では肥満のみでは有意なCKDリスクの増加は認めなかった（OR 1.01, 95%信頼区間0.71-1.44)。

結論：代謝異常を合併しない肥満者において、男性ではCKD有病リスクが有意に高かったが、女性では有意な上昇は認めなかった。

キーワード：腹部肥満、慢性腎臓病、メタボリックシンドローム、性差
